# Participation 3.0 in the implementation of the energy transition—Components and effectiveness of an interactive dialogue tool (Vision:En 2040)

**DOI:** 10.1371/journal.pone.0299270

**Published:** 2024-03-04

**Authors:** Julia Thiele, Julia Wiehe, Christina von Haaren

**Affiliations:** 1 Institute of Environmental Planning, Leibniz Universität Hannover, Hanover, Lower Saxony, Germany; 2 Department for specialist information, Kompetenzzentrum Naturschutz und Energiewende KNE gGmbH, Berlin, Germany; Izmir Katip Celebi University: Izmir Katip Celebi Universitesi, TURKEY

## Abstract

The allocation of renewable energy plants, especially wind turbines, is stagnating in Germany. Although the citizens approve of the energy transition, they resist concrete local projects. In recent years, research has shown that interactive map applications support participatory planning through motivation, social interaction, and knowledge transfer. We aim to reduce biases against renewable energy (RE) and support informed decision making while accepting local responsibility. We hypothesized that finding a new gamified participation format, based on behavioral mechanisms, would strengthen the empowerment of people. To this end, we designed a dialogue tool and participation format, ’Vision:En 2040’, which combines: (i) a precise target electricity yield, (ii) an interactive map showing results of people’s actions, information about environmental impacts, and (iii) game rules which foster cooperation. In facilitated workshops, participants simulate the allocation of wind and photovoltaic power plants in their municipality to achieve a target electricity yield. The developed tool is based on methods of environmental planning and geoinformatics. ’Vision:En 2040’ was systematically tested with a technical test and a pre-test. In addition, its impact on participants was assessed through surveys and qualitative content analysis. The evaluation results show that the tool can influence the acceptance of the energy transition in terms of attitude. Through ’Vision:En 2040’, participants became aware of the community’s responsibility in the energy transition and expanded their knowledge. In addition, decision makers used the workshop results to plan RE sites. Our results indicate that ’Vision:En 2040’ is helpful for informal citizen participation in accelerating the energy transition.

## 1. Introduction

Achieving the 1.5°C target of the international climate change agreements (COP 21) is a global challenge today. According to the Intergovernmental Panel on Climate Change (IPCC) modeling, this goal would require a net global reduction in anthropogenic carbon dioxide (CO2) emissions of about 45% from 2010 levels by 2030 to reach net zero around 2050 [1]. Reducing CO2 emissions requires a shift from fossil fuels to renewable energy (RE). According to the German Renewable Energy Sources Act (EEG), the share of electricity from renewable sources in gross electricity consumption must increase to at least 80% by 2030 (§ 1 EEG [§ 1, 2]). In 2021, Germany’s share of RE in gross final energy consumption was 19.2% [3]. Accordingly, the share of RE must increase massively over the next eight years to meet the 2030 target [2]. Apart from wind power, solar energy is the only sustainable energy source available in the country on a system-relevant scale and offers a sufficiently efficient greenhouse gas reduction balance per unit area [4]. Therefore, in 2022, the legislature enacted new laws (e.g. the Wind-on-Land Act) to dramatically increase wind and solar energy.

Most German citizens support the energy transition [5]. In practice, however, the allocation of RE has been hampered by citizen protests and legal action against planning approvals [6–8]. Concrete local planning influences the acceptance of renewable energy systems: Even if acceptance was high before the start of planning, for instance of local wind farms, it decreases when the planning process begins [9]. Residents’ desire for greater and earlier involvement in the planning process [10, 11]. In addition, a study found that fear of potential health impacts, insufficient consideration of nature and landscape conservation, and insufficient contribution to climate protection are the most common arguments used by critics [12]. Media narratives about wind power expansion also reinforce the apparent conflict between energy system transformation and conservation [13]. In general, the following factors influence the acceptance of infrastructure projects: The number of interested and informed participants, the collaborative processes, the spatial delineation of problems, and the transparency of different stakeholder interests. It is also hypothesized that acceptance will increase if people are made aware of their local responsibility for global challenges [14]. As local protests become more entrenched [12], it can be assumed that previous forms of participatory planning and permitting have not fostered local acceptance of RE allocation or socially responsible decision-making.

Newer participatory approaches combine interactive map applications and gamification elements to raise awareness of environmental issues, share knowledge transparently and collaboratively, and encourage behavior change [15–17]. For example, Shrestha et al. [18] conducted "planning game workshops" using interactive maps on a "map table" to explore social learning and knowledge acquisition. Furthermore, Flacke and Boer [19] developed an interactive planning support system for the energy transition process. In their research, participants were able to locate RE plants on an interactive map. However, the participants were not able to calculate how much their planning had contributed to the overall demand for RE in the Netherlands or to the RE expansion target of the specific municipality. Lanezki et al. [20] designed a serious game to communicate knowledge about the energy transition to citizens. They concluded that the energy transition at the local level is a suitable topic for gamification and that it can be used for citizen participation.

Gamification and serious games have been used to break down habits and inhibitions and create behavioral change [20–23]. It is defined as "the use of game design elements in non-game contexts" [24]. Gamification can promote autonomous motivation by providing users with a sense of the three psychological needs of autonomy, competence, and relatedness [25, 26]. Autonomous motivation has a longer lasting positive effect and is therefore the desired type compared to controlled motivation [27]. Gamification by itself does not affect autonomous motivation, behavior, or cognitive learning [28, 29]. To support autonomous motivation, according to self-determination theory, it should address the three psychological needs, for example, by creating motivating experiences of achievable goals and social interactions in a flexible system [27]. Capellán-Pérez et al. [23] developed a participatory simulation game to create climate change strategies and showed that its use stimulated discussions on critical issues. These approaches, which integrate interactive map applications and gamification elements, have been developed for collaborative, spatial planning to increase acceptance in planning processes. They go beyond simple information sessions and require active participation. Gamification and the use of map tables could therefore help to reduce the acceptance problems described above. So far, there is no digital dialogue tool in Germany that implements these approaches as an informal participation tool to discuss the energy transition on a local level together with decision makers. Furthermore, the integration of behavioral mechanisms, which have been found to be relevant for environmental planning [30], has not been tested in a participation tool.

With this in mind, we aim to reduce conflicts and increase the acceptability of RE through a new gamified participatory dialogue tool. We hypothesized that the following factors are central to this goal: raising people’s awareness of their responsibility to mitigate climate change, providing information about the limits of sustainable allocation of RE, and new gamified participation formats. In addition, we used the following behavioral mechanisms for the operational configuration of the participation concept: slowing down decisions by asking participants to explain them, competition between groups, downscaling of big problems to the local level, disclosure of information, codification of information/ease of comparison and tangible results [according to 30]. In combination, these factors should empower citizens and improve decision-making and acceptance in local contexts.

For this purpose, we designed and tested a dialogue tool called ’Vision:En 2040’. ’Vision:En 2040’ combines the following aspects in order to mitigate the proven arguments of critics, to promote a factual dialogue and to create a transparent opportunity for participation: (i) a target electricity yield that is downscaled from the national to the local level and represents local responsibility for achieving the national climate goals, (ii) an interactive interface that shows immediate results of people’s actions, and provides information on the potential impacts of RE on biodiversity and ecosystem services, and (iii) game rules that encourage both cooperation and competition among players. The digital dialogue tool and its participation concept were developed for application at the municipal level, where people can cooperatively simulate scenarios for local RE allocation. The results can subsequently be adopted by decision makers.

In developing ’Vision:En 2040’, we have explored how scientific findings can be transferred into practice to promote acceptance and decisions for an energy transition. Our research questions were whether ’Vision:En 2040’ is able to (i) change attitudes towards the energy transition and the extension of renewable energy plants locally, (ii) make citizens aware of their own local responsibility in the energy transition process, and (iii) awaken understanding for other opinions in their own municipality.

We structured this paper as follows: In section 2, we describe the conceptual framework of ’Vision:En 2040’ and present the interface and functionalities of the digital dialogue tool. The workshop concept is also highlighted in section 2, followed by a demonstration of the implementation process and the quantitative as well as qualitative evaluation of ’Vision:En 2040’. Section 3 shows the evaluation results from a public workshop. The paper closes with a discussion and conclusion (section 4 and 5), in which we reflect on the strengths and limitations of ’Vision:En 2040’ against the background of the research questions.

## 2. Development and implementation of ‘Vision:En 2040’

### 2.1 Conceptual framework of ‘Vision:En 2040’

’Vision:En 2040’ aims to empower citizens and improve the acceptance of RE systems in the local context. We define acceptance according to Schweizer-Ries [31]: "The acceptance of renewable energy technologies represents the positive, relatively constant result over time of an appreciation process of the respective technology by an acceptance subject that is linked to certain framework conditions (= evaluation level). This positive evaluation can also be accompanied by actions corresponding to this evaluation judgment and the perceived framework for action (= action level)". According to this definition, acceptance can be represented by the levels "evaluation (positive to negative)" and "action (active to passive)”.

Knowledge and information also promote acceptance of RE expansion [32, 33]. Acceptance increases with the level of information and knowledge about RE [34]. In a literature review, Schauff [33] found that people who are familiar with the topic of wind energy tend to be more accepting. Fostering interest and knowledge, as well as providing understandable and accessible information, are levers for building acceptance. In addition, specific local information can mitigate some irrational and counterproductive behavioral mechanisms in decision making [30]. In order to achieve an increase in acceptance through knowledge and information, the source of information must be independent, objective, knowledgeable, and reliable [35]. Information should be communicated in a transparent, understandable and targeted manner with relevant and consistent content.

**Fig 1:**
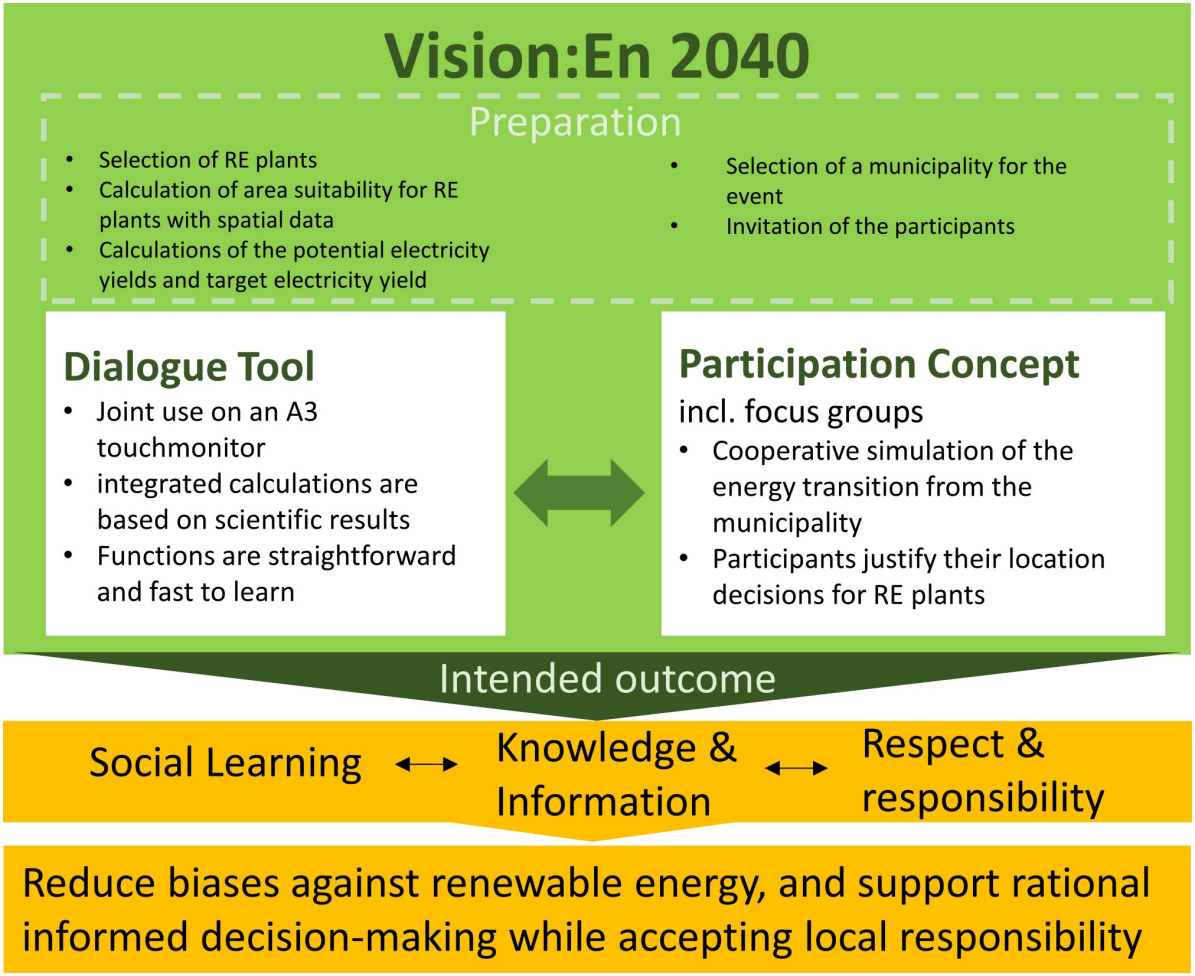
Conceptual framework of the interactive dialogue tool for renewable energy allocation ‘Vision:En 2040’ [16, 18].

With this in mind, we have developed the following concept for a dialogue and participation concept that counteracts prejudices and supports people in making decisions about "their" future energy landscape (Fig 1).

Participants receive an introductory presentation at the beginning of the workshop, in which the key points of the energy transition are presented. Subsequently, participants use the dialogue tool to simulate the energy transition for their municipality and exchange arguments about the siting of RE plants. Each placement of a RE plant is justified. The tool and its workshop enable social interaction that promotes collaborative knowledge production and interpersonal communication [36]. It provides a space for social learning that encourages understanding of different positions and reflection on one’s own statements and actions [37, 38].

Social learning encompasses learning in and with social groups through interactions [39] and can be helpful for successful environmental management [40]. A social learning process is intended to stimulate a change in understanding among the individuals involved, and this change should extend beyond the individual [41]. The results of a ’Vision:En 2040’ workshop should therefore have an impact beyond the participants and be relevant to decision makers. In social learning, different actors intrinsically contribute their own arguments, which arise from autonomous motivation and can be supported by gamification. The digital dialogue tool promotes dialogue processes through gamification elements and provides knowledge by:

- transparent visualization of suitable areas that can be used in a way that is compatible with people and nature on an interactive map. Participants can use it to plan the distribution of RE plants in a way that is compatible with the landscape (cf. criticism of opponents, chap. 1) and to identify the potential of the municipality. Since the processed information is available locally and concretely for the known territory, it influences the actors’ decisions in a particular way [42].
- showing potential electricity yields for plant placements.
- presentation of a target electricity yield that the municipality would have to achieve in order to meet Germany’s climate protection targets (cf. criticism of opponents, chap. 1). The target year for the RE allocation simulation is 2040 to highlight the current need for action today [43, 44]. This communicates the municipality’s responsibility to achieve a 100% renewable energy supply.

The dialogue tool with integrated gamification elements is operated by the workshop participants via a touch monitor, as studies have shown that the use of "map tables" can enable and promote participatory planning [17, 19, 45].

### 2.2 ‘Vision:En 2040‘: Digital dialogue tool and participation concept

#### 2.2.1 Participation concept

**Fig 2:**
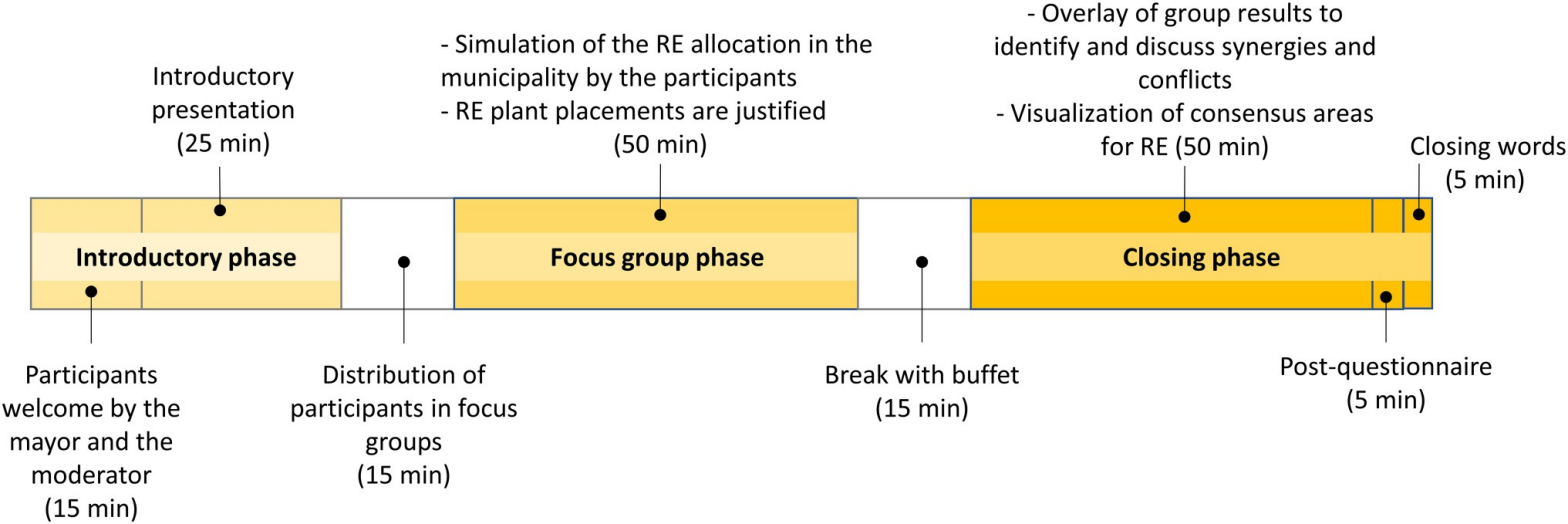
The three phases of the participation concept: Introductory phase, focus group phase, and closing phase.

According to the conceptual framework (Fig 1), the dialogue tool is embedded in a three-hour evening workshop [19], which is divided into three phases (Fig 2). The target group of ’Vision:En 2040’ includes all stakeholders of a municipality, such as interested individuals and representatives of local politics and public administration. Participants are invited through the local press and social media platforms. Upon entry, participants receive a name tag with a colored dot that is randomly assigned to the composition of the focus group.

In a 40-minute introductory phase (Fig 2), participants are welcomed by the mayor of their municipality. Participants then receive an overview of the status of the energy transition and the environmental impacts of different RE systems. Health impacts, a key point of criticism from opponents [12], are also addressed in this introductory input.

In a subsequent focus group phase, the participants collaboratively simulate the RE allocation in their municipality. A study showed that the speaking part is balanced with six people in a focus group [46]. Since six touch monitors are available, a total of 36 people can participate in a workshop. Participation will be determined by lottery if more people register. The focus groups are facilitated by a moderator who supports the use of the dialogue tool [19].

After a 15-minute break, the results of the focus groups are presented in a final plenary session. For this purpose, the results of the focus groups can be overlaid in the dialogue tool to identify and discuss synergies and conflicts of the different simulations. Areas of consensus can be identified and made available to decision makers.

#### 2.2.2 Dialogue tool: Main components and functions of the user interface

**Fig 3:**
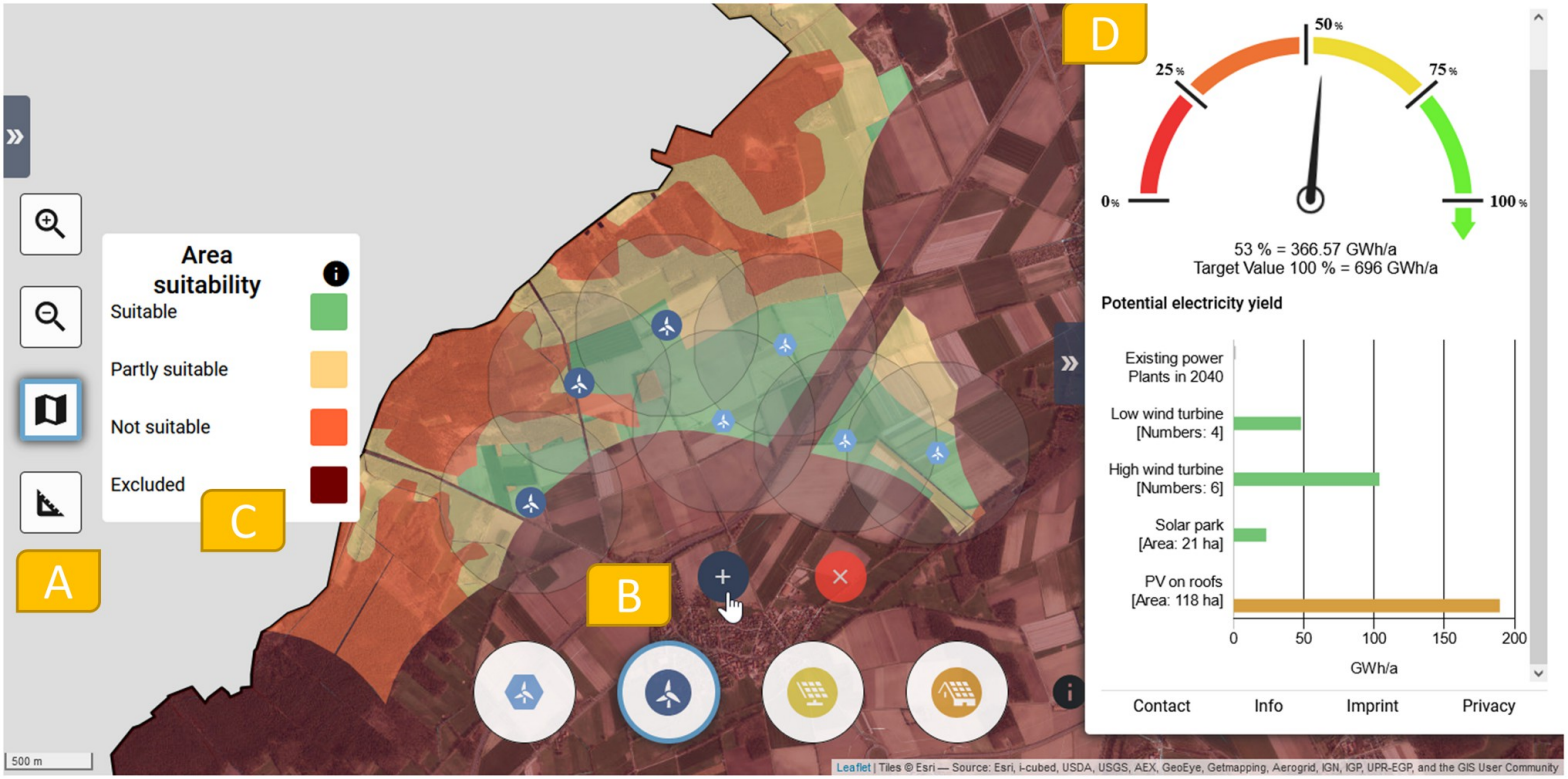
Screenshot of the dialogue tool interface during the focus group. (A) On the left side of the screen, the user can zoom into the interactive map as well as activate an aerial view [47] and a measurement tool. (B) The higher wind turbine has been selected, (C) and its classes of area suitability are displayed. On the left side of the screen is a legend explaining the classes of area suitability. (D) On the right side of the screen, the Ener_geter, a kind of tachometer, shows the target electricity yield of the municipality and the extent to which this has been achieved by the placed renewable energy plants.

*2*.*2*.*2*.*1 User interface of the RE plant distribution in focus groups*. The dialogue tool’s interface displays the municipality area as a digital map in the center screen (Fig 3). On the left side of the screen, the user can zoom in or out, activate an aerial view, and activate a measurement tool to measure, for example, the distance between a settlement and a placed wind turbine. In Fig 3A, the aerial view is active.

In the lower part of the screen, icons of the RE plant types are integrated into the dialogue tool: a lower wind turbine (nominal power 4.2 MW, hub height 130m, rotor diameter 138.25m), a higher wind turbine (nominal power 5.5 MW, hub height 180m, rotor diameter 160m), solar farms, and rooftop PV. Information on the different types of RE plants can be accessed by clicking on the Information button.

When touching an icon of a RE plant type, the municipality area changes its color to the area suitability classes of the plant type [48]. Area suitability describes the potential of a location for wind energy or solar parks, taking into account nature conservation and human well-being [49]. (calculation background see S1 Text). It is divided into four classes: suitable, partly suitable, not suitable, and excluded (Table 1).

**Table 1:** Definition of the four area suitability classes, displayed in the dialogue tool as a gamification element, if a wind turbine or open space photovoltaic systems is to be placed in the municipality [50].

Classes of area suitability	Description
suitable	According to consistent nationwide evaluation criteria, the area can be used in a way compatible with human well-being and nature.
partly suitable	The area can be used in a way that is compatible with humans and nature, with restrictions, according to consistent nationwide evaluation criteria.
not suitable	The use is incompatible with human well-being and nature due to legal regulations and requirements.
excluded	The use is not compatible with humans and nature for technical reasons, legal regulations and requirements.

Once a wind turbine has been placed, a light gray ring visualizes the minimum distance to be maintained to another wind turbine (3.25 times the rotor diameter). If the turbine is placed outside of a suitable or partly suitable area, a warning message is displayed that this location is not compatible with humans and nature. By selecting the placed RE plant, the user receives information about the location of the plant, such as its protection status. To add a solar park, an area is digitized using support points at the corners and edges.

Different rules apply to the use of rooftop PV, as almost all roofs can be used without conflict from a conservation perspective. Accordingly, no suitability classes are displayed. To adjust the amount of PV on their roofs, participants use a slider to select a percentage of the rooftop PV potential to be implemented in their simulation. The settlement area of the community is colored darker, the more usable roof area is to be covered with modules.

The dialogue tool calculates the potential annual electricity yield of the placed RE plants (calculation background see S2 Text). In the ’Ener_geter’, a kind of tachometer (Fig 3D), the tool compares the potential annual electricity yield of all assigned RE plants with the target electricity yield (target value) in percent. The target electricity yield for 2040 describes its contribution to the success of the energy transition and is derived for each municipality from nationwide development scenarios [51]. Due to the sensitivity of the landscape and the settlement structure in Germany, the usable area potential is not evenly distributed. Therefore, the target electricity yield of a municipality does not match its energy needs. If all municipalities were to achieve their tailor-made target electricity yield according to their potential, the nationwide goal of an energy transition that is compatible with human well-being and nature would be achieved. The startup setting of the Ener_geter’s corresponds to the percentage of electricity from RE plants that could still be in operation in 2040 relative to the target electricity yield of the municipality (calculation background see S3 Text).

Below the Ener_geter, a bar graph shows how much electricity can be expected from each type of renewable energy system and how many wind turbines and how much area for solar parks the focus group participants included in their simulation (Fig 3D).

**Fig 4:**
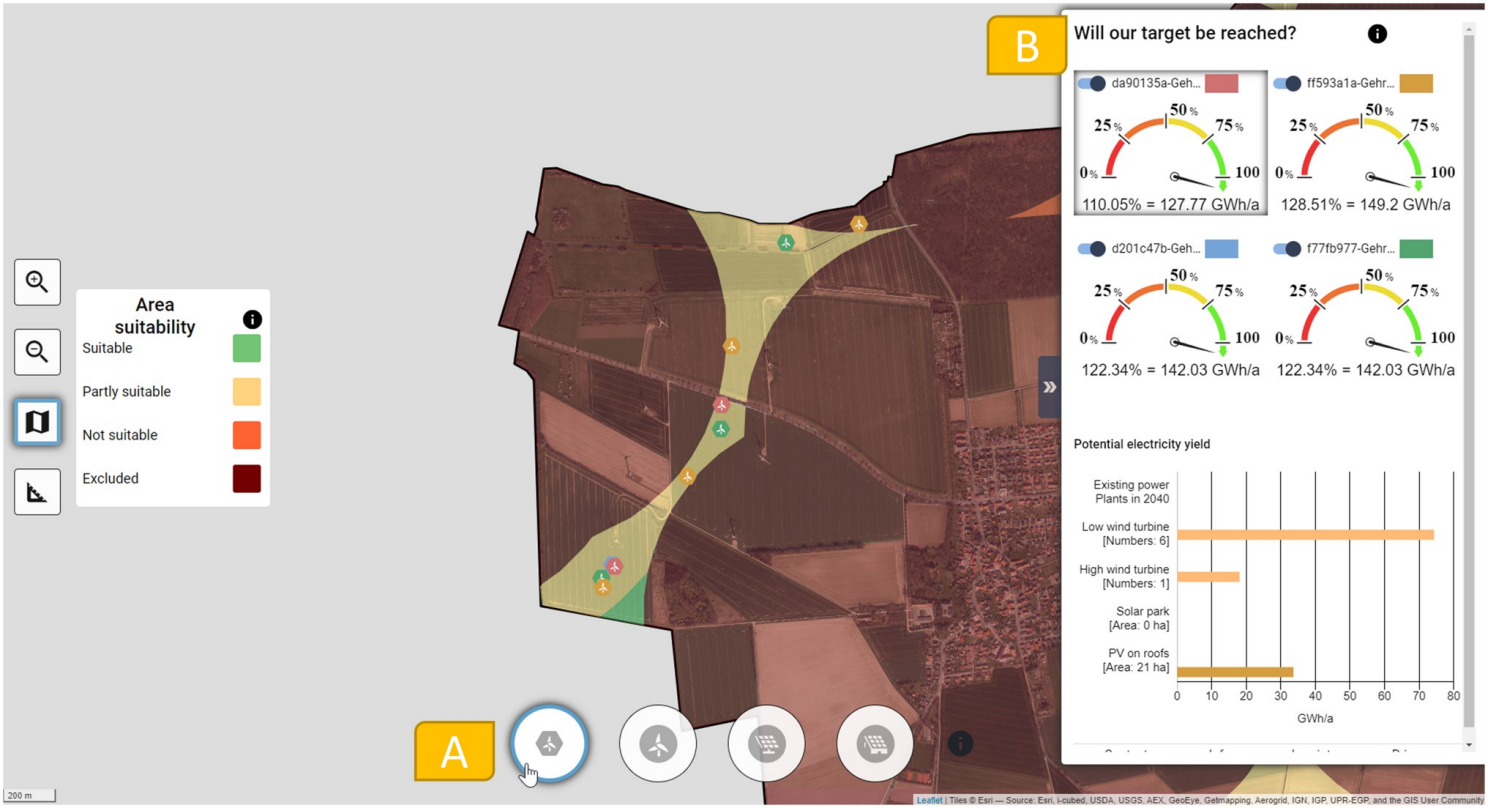
Screenshot of the user interface of the dialogue tool during the closing phase. An aerial view is shown in the background [47].

*2*.*2*.*2*.*2 User interface for overlaying focus group simulations in the closing phase*. The user interface to overlay the group results in the closing phase of the workshop is similarly structured and allows the comparison of the simulation results (Fig 4).

The facilitator selects a RE plant icon (Fig 4A), and the dialogue tool displays the overlay of the simulations from all focus groups. Each focus group has been assigned a color for this purpose. Fig 4 visualizes the focus group results for the smaller wind turbine. With this overlaid representation, hotspots can be quickly identified to discuss potential conflicts and synergies for the focus groups’ siting choices. The goal here can be to jointly define consensus areas for this plant type and to share both plant locations and potential consensus areas with decision makers. The focus group Ener_geters are displayed side-by-side on the right-hand side of the screen (Fig 4B) so that their results can be compared simultaneously at a glance.

### 2.3 Implementation and evaluation of ‘Vision:En 2040’

#### 2.3.1 Study area

**Fig 5:**
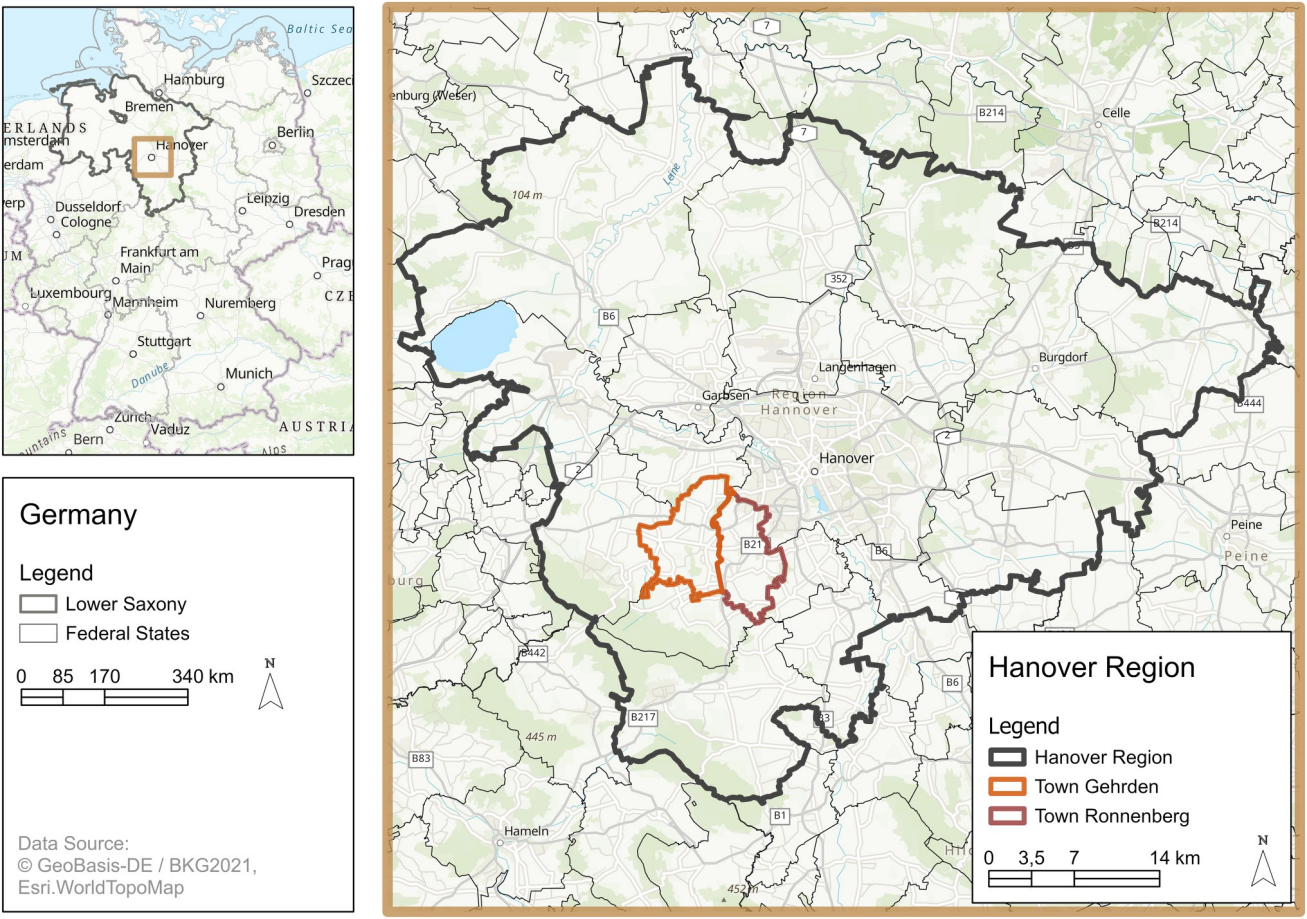
Study area of ‘Vision:En 2040’. The dialogue tool can be used in every municipality and town in the Hanover Region and has been used for the first time in Gehrden and Ronnenberg.

’Vision:En 2040’ was initially developed for the Hanover region (NUTS 3 region), a municipal association of 21 cities and municipalities in the state of Lower Saxony, in the northwest of Germany (Fig 5).

The Climate Protection Act of Lower Saxony (NKlimaG [52]) aims towards a complete coverage of the energy demand by RE by the year 2040 (§ 3 para. 3 NKlimaG). Lower Saxony currently has the largest installed capacity for onshore wind energy in Germany and is in the upper midfield for the newly installed photovoltaic capacity [53]. Compared to the rest of Lower Saxony, the Hanover Region ranks in the upper midfield for both onshore wind and solar energy expansion [54].

#### 2.3.2 Technical test, pre-test and public workshop

The functionality and usability of the dialogue tool prototype were tested during the test phase using a systematic test procedure. The systematic test procedure included a technical test and a pre-test.

**Fig 6:**
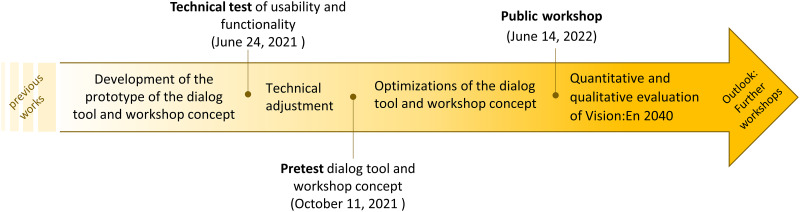
Development and testing phases of ‘Vision:En 2040’: Technical test, pre-test and public workshop.

The technical test was conducted on June 24, 2021 with 17 students from Leibniz University of Hanover (Fig 6), who were divided into three groups for the technical test. During the technical testing of the dialogue tool, the students worked on test tasks to evaluate the usability and functionality of the tool. Realistic testing tasks are used in software development as a feedback technique to test the key functions of the system [55], to focus the testing, and to collect quantitative values [56]. After each test task, students rated the usability and functionality in a questionnaire.

The whole concept of ’Vision:En 2040’ was tested on October 11, 2021 in the festival hall of the town Gehrden. For the pre-test, local mayors, employees of planning offices, the town council, nature conservation associations, and the energy sector were invited by the climate protection manager and the climate protection agency Region Hannover. 21 people participated in the pre-test under conditions of the COVID-19 pandemic. The pre-test was then evaluated quantitatively and qualitatively. The results of the technical test and the pre-test were used to adapt the dialogue tool and the workshop concept.

After optimization, a public workshop was held in Ronnenberg on June 14, 2022. The workshop was announced publicly so that every resident of Ronnenberg had the opportunity to register. The public workshop was quantitatively and qualitatively evaluated.

#### 2.3.3 Quantitative and qualitative evaluation of ‘Vision:En 2040’

*2*.*3*.*3*.*1 Structure of the standardized questionnaire*. In order to investigate whether ’Vision:En 2040’ can increase the acceptance of local RE sites and initiate dialogue processes in the municipality, and what application possibilities and optimization potentials exist, the participants received a post-session questionnaire. It included closed, hybrid and open-ended questions and could be answered within five minutes. Respondents rated the statements in three question matrices on a 4-point Likert scale ("agree", "tend to agree", "tend to disagree" and "disagree") with an additional "not specified" option. In this scale, respondents had to choose a response direction, but had the option of choosing the non-content response category "not specified" [57]. Comparable studies have used this approach [5, 58–60].

In the first question matrix, statements about ’Vision:En 2040’ were evaluated regarding the respondent’s attitude towards the energy transition. For example, the statement "Vision:En has shown me that it is possible to locate RE in the municipality while taking into account nature protection and human well-being" was included in the questionnaire.

In the following question matrix, the participants mainly evaluated the usability of the tool. After this matrix of questions, an open-ended question allowed participants to make suggestions for improving usability and functionality, as well as to express their wishes for additional features.

The final question matrix included statements about the concept of participation and was divided into three parts: Statements about the introductory phase, the focus group phase, and about the closing phase. In this question matrix, for example, participants rated the time frame of each event phase. The questionnaire ended with three open-ended questions. Here, participants could list suggestions for improvement and ideas for the schedule.

Sociodemographic characteristics were not collected in the five-minute survey because participants were not to be detained any longer after the workshop. The handwritten questionnaires were digitized in the online survey tool LimeSurvey (version 3.28.5) and then analyzed descriptively in Excel 2019.

**Fig 7:**
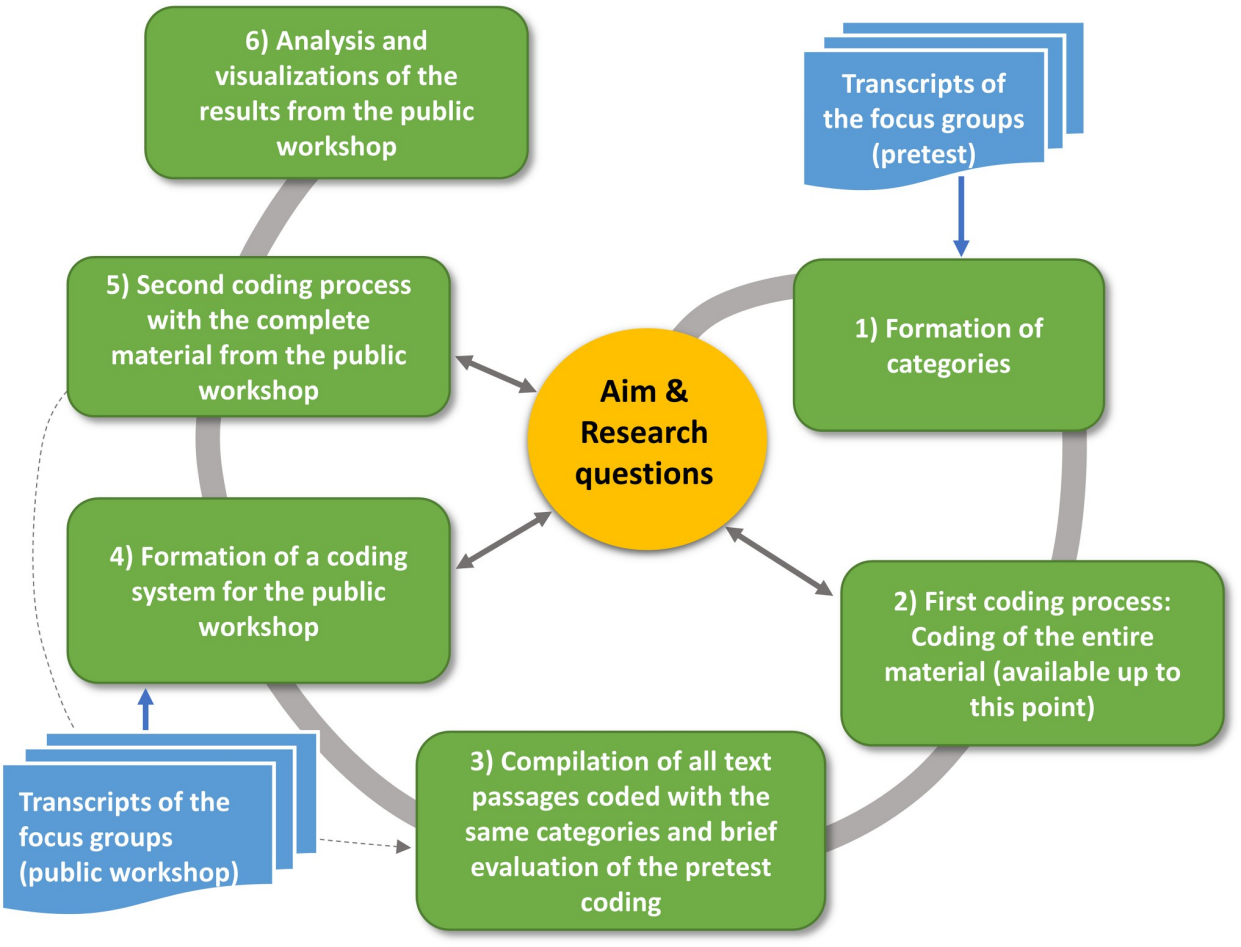
Six phases of the content-structured content analysis to analyze the focus groups (based on Kuckartz [62]).

*2*.*3*.*3*.*2 Qualitative evaluation of the focus groups*. In addition, we examined the discussion process of the focus groups with a structuring qualitative content analysis. For the qualitative content analysis, the screens and conversations of the focus groups were recorded. The transcription and subsequent steps were performed in MAXQDA software (version, 2022.0.0), using the content-semantic transcription rules according to Dresing [61]. The workflow of the qualitative content analysis is divided into six phases (Fig 7), based on Kuckartz [62]:

For the qualitative content analysis, deductive categories were initially developed in the first phase, which were supplemented by inductively formed categories through a coding of test material. In the third phase, the transcripts of the pre-test focus groups were coded and a brief analysis was conducted. In the fifth phase, a coding system for the public event was created. For this purpose, the experiences from the coding process of the pre-test material were used on the one hand, and a coding system developed independently by students of the Leibniz University of Hanover was used on the other hand, since the creation of a coding system should be carried out independently by several people [62]. In order to combine the coding systems, three transcripts of the public workshop were coded in order to avoid having to create additional categories during the subsequent coding of the entire material.

In the final analysis, we contrasted the pros and cons of the placed RE plants to show which type of RE plant received the least or most pros or cons from the participants. For this category-based evaluation, the appropriate categories were selected and transferred to a table so that each statement could be presented in its own words or with a greatly reduced quote. Similar or comparable statements were listed in order to determine which arguments were most frequently mentioned in the focus groups.

The presentation of the results in this paper focuses on the public workshop in Ronnenberg, since optimizations of the dialogue tool and the evaluation methods were used here (e.g. a coding system for qualitative content analysis that was revised after the pre-test).

## 3. Results

### 3.1 Results of the questionnaire evaluation

The public workshop in Ronnenberg was attended by 24 participants from agriculture, energy, administration and politics, who were divided into five focus groups. All focus groups achieved the calculated target electricity yield for the year 2040. One focus group even simulated an expansion plan that exceeded the target electricity yield by 99%. At the end of the event, 21 participants completed the survey.

**Fig 8:**
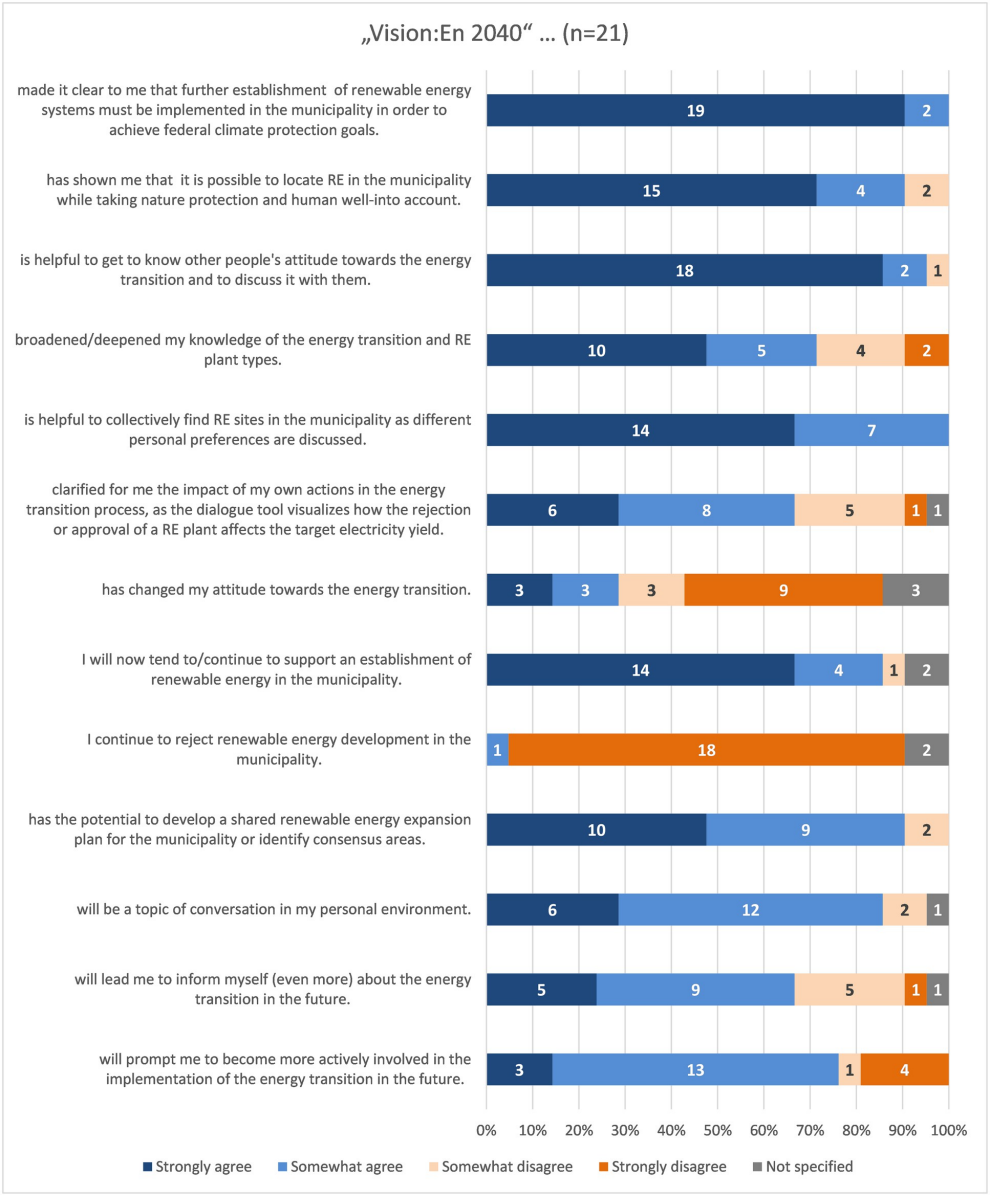
Evaluation of the question matrix with statements about ‘Vision:En 2040’.

All participants agreed or tended to agree with the statement, "Vision:En 2040 made it clear to me that further establishment of renewable energy systems must be implemented in the municipality in order to achieve federal climate protection goals" (Fig 8). When pre-tested, 90% also agreed with this statement.

**Fig 9:**
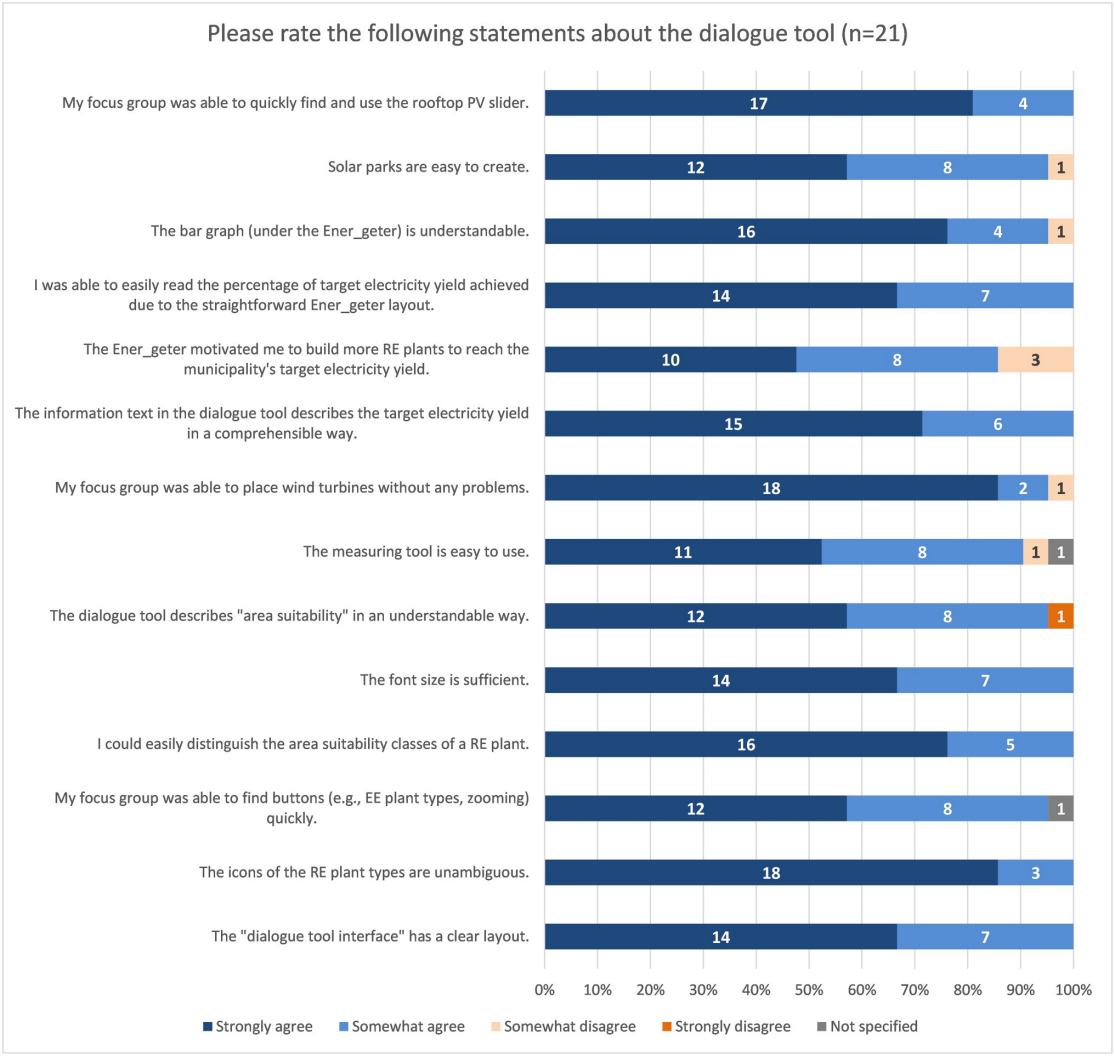
Evaluation of the question matrix regarding the usability of the dialogue tool.

For 100% of the respondents, ’Vision:En 2040’ was helpful to jointly find RE sites in the municipality, as different personal attitudes were discussed. In the pre-test, 90% of the respondents agreed with this statement, so again a similar response pattern was found. Furthermore, ’Vision:En 2040’ showed that 90% of the respondents believe that it is possible to locate RE in Ronnenberg while respecting nature and human well-being (Fig 9). For 90% of the respondents, ’Vision:En 2040’ also offers the potential to develop a joint allocation plan for renewable energies in the municipality or to identify consensus sites. Over 95% of pre-test respondents indicated that ’Vision:En 2040’ will be a topic of personal discussion. In addition, 67% of respondents said that ’Vision:En 2040’ will encourage them to learn (even more) about the energy transition in the future (Fig 8). On top of that, 76% of the respondents even stated that they plan to become more actively involved in implementing the energy transition in the future. Agreement with these two statements was lower in the pre-test: 57% and 67%, respectively.

The evaluation results of the usability statements showed positive results: 100% of the respondents agreed that 7 of the 14 statements were true or somewhat true (Fig 9). In the pre-test, the usability statements received lower agreement rates: 85% of the respondents "strongly agree" or "somewhat agree" with 9 of the 14 statements.

For example, respondents found the font size in the dialogue tool to be sufficient, they were able to distinguish well between the classes of area suitability of a RE installation, and they found the layout of the dialogue tool interface to be clear (Fig 9). The information text was also easy for respondents to understand, and the rooftop PV usage slider was easy to find and use. Only one respondent considered that the dialogue tool did not describe "area suitability" (cf. chap. 2.2.2.1) in an understandable way.

In the open question about suggestions for improvement, one person mentioned the following: "When setting the icons, e.g. wind energy, give an error message when setting incorrectly. Some people did not know that the circles from the wind turbine are allowed to overlap". In the pre-test, two people commented that they could not distinguish the area suitability classes of a RE plant very well. This was optimized and the respondents did not mention this again.

**Fig 10:**
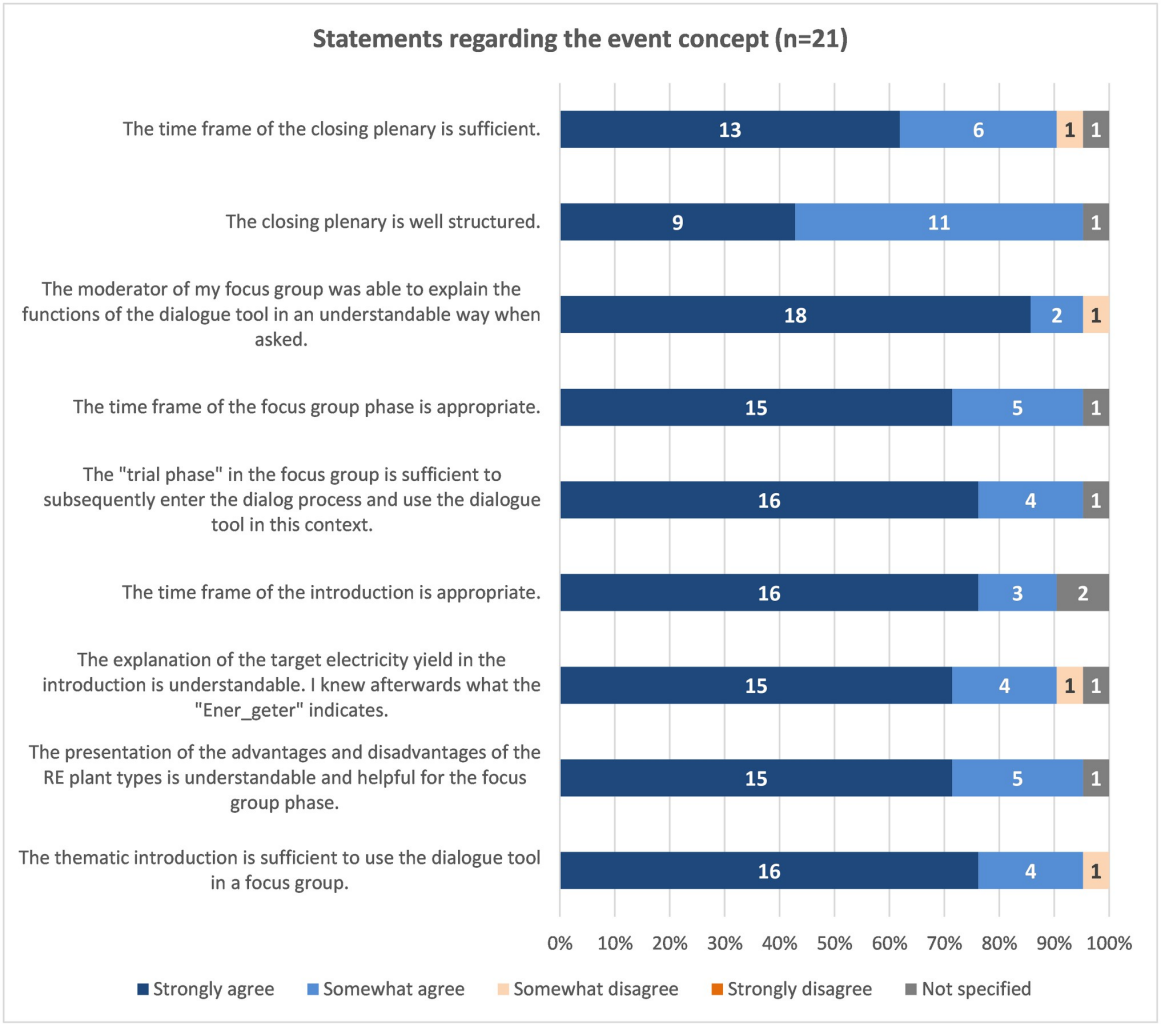
Evaluation of the question matrix regarding the participation concept.

The answer option "rather not correct" was not marked in the question matrix for any of the participation concept statements. At least 90% of the respondents agreed or tended to agree with the statements questioned (Fig 10). In the pre-test, agreement was mostly above 80%.

The answer option "rather not correct" was not marked in the question matrix for any of the participation concept statements. For example, 90% of respondents rated the implementation timeframe as reasonable (Fig 10). Only one person stated that the introduction should be extended so that the tool could be used for simulation afterwards. Respondents felt that the closing plenary was well structured and that the time frame was appropriate. One person mentioned two suggestions for the introductory phase: "The importance of energy storage (hydrogen) was not conveyed", and "Target 2040 must be brought forward to 2035".

**Fig 11:**
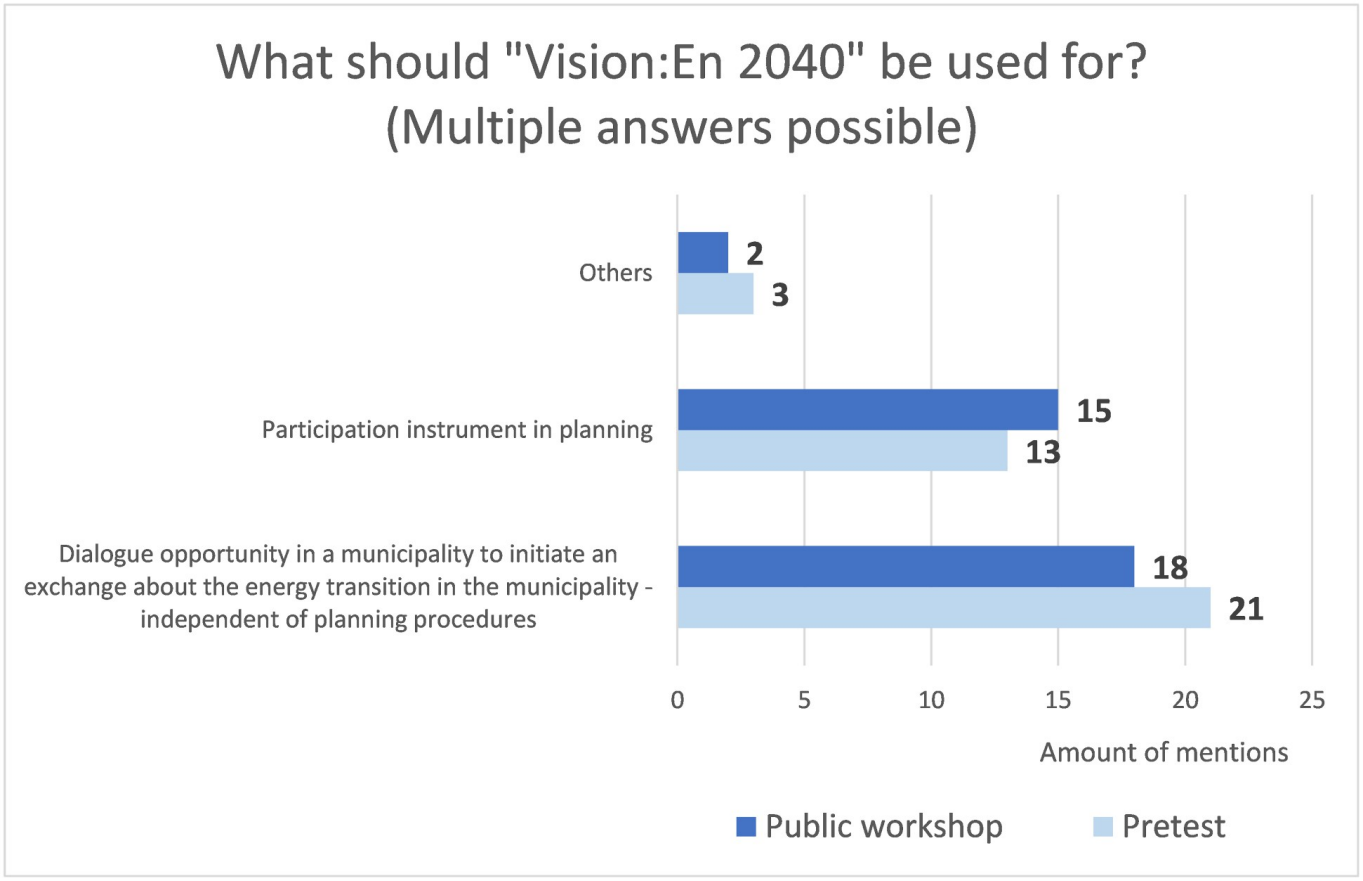
Possible uses of ‘Vision:En 2040’. Answers to the question what ‘Vision:En 2040’ should be used for–multiple answers were possible (pre-test, n = 21; public workshop n = 21).

The majority of respondents (86%) stated that ’Vision:En 2040’ should be used as an opportunity for dialogue in the community to initiate an exchange about the energy transition in the municipality–independent of planning processes. In fact, this answer option was checked by all participants in the pre-test (Fig 11). 71% of the respondents see ’Vision:En 2040’ as a participation tool in planning processes and two people mentioned ’schools’ as a possible use under ’other’. On average, 1.7 answers were checked (1.8 in the pre-test).

### 3.2 Results of the quantitative evaluation of the focus groups

**Fig 12:**
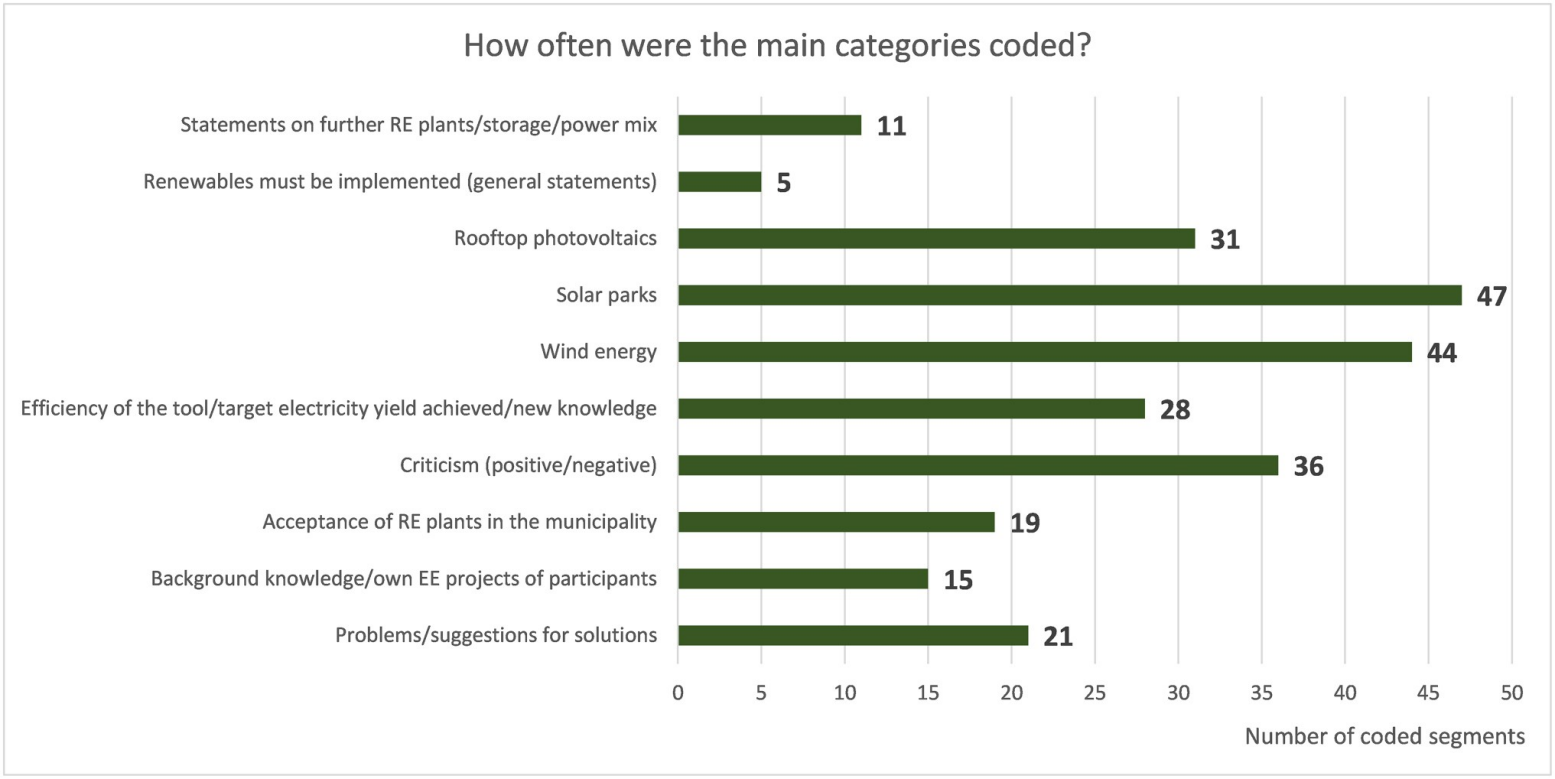
Focus group coding. Overview of the frequency of the ten main categories, including their subcategories, across the five focus group transcripts.

A total of 257 text segments were coded in the transcripts of the five focus groups of the public workshop in Ronnenberg. The main category "solar parks" with its subcategories "arguments against placement" and "arguments for placement" was coded most often, followed by the main category "wind energy" (Fig 12). Participants in the focus group discussions also expressed positive and negative criticism of society, the dialogue tool, or legal requirements.

The analysis shows that the course of the discussion was different in each focus group. For example, while two focus groups were discussing problems, proposed solutions, and the allocation of wind turbines at the beginning of the simulation, another focus group was discussing the setting of the PV slider in detail. In all focus groups, the four types of RE systems integrated into the dialogue tool were discussed and placed, and the electricity yields achieved were compared with the Ener_geter status. The following section presents the discussion content of the coded segments of the main category "wind energy".

In all focus groups, there were more arguments in favor of siting wind turbines (82%) than against siting (18%), and both types of wind turbines were included in the simulation. Participants agreed that all suitable areas should be fully utilized and raised no objections to the siting of wind turbines in suitable areas. Thus, the visualization of area suitability in the dialogue tool had a strong influence on the siting of wind turbines. In some cases, participants fully utilized areas that were partly suitable for wind turbine siting (cf. chapter 2.2.2.1). Other arguments for integrating wind turbines included the fact that there are already wind turbines in the area, that farmers are likely to make their land available, or that people do not live too close to the selected site. The analysis of the placement discussions showed that focus group members picked up on each other’s arguments, thus passing on information and knowledge. The exchange of views on the placement of wind turbines strengthened the participants’ positive attitude towards local RE development.

## 4. Discussion

Based on interactive participation tools [17, 23, 45, 63], including behavioral mechanisms and existing energy transition scenarios [4, 51, 64], we created a participatory dialogue tool for renewable energy allocation (’Vision:En 2040’) to support the local energy transition. The use of the tool in the context of moderated participatory processes reduces conflicts and increases local acceptance for the installation of RE systems by raising people’s awareness of their responsibility for climate protection and providing information on sustainable locations for RE systems.

With the qualitative and quantitative evaluation of ’Vision:En 2040’ we were able to show that ’Vision:En 2040’ has put scientific knowledge about the energy transition into practice. Scientific knowledge was disseminated through both the workshop concept and the dialogue tool. For example, in the first phase of the event, an introductory presentation provided participants with general information about the energy transition and the impact of RE installations (Fig 2). From the participants’ perspective, this information was presented in an understandable, target-group-oriented, and transparent manner [30], factors that positively influence recruitment acceptance (see research question 1, introduction).

In addition, the visualization of area suitability classes in the dialogue tool illustrated which areas in their municipality could be used for RE allocation in a way that is compatible with human well-being and nature. This could help to an increase in acceptance, as Hübner et al. also showed that RE installations built in a way that is compatible with nature and the landscape are more accepted [65, 66].

’Vision:En 2040’ communicates local responsibility in the energy transition by using gamification elements. For the participants, both the visualization of the target electricity yield on the Ener_geter and the display of suitable areas emphasized the potential and responsibility of their municipality in the RE installation process. This was also confirmed by the standardized survey: ’Vision:En 2040’ showed the participants that a further construction of RE plants in the municipality is necessary to meet the projected nationwide energy demand from renewables (see research question 2, introduction). The Ener_geter status motivated the discussants to include as many RE plants as necessary to meet or exceed the target electricity yield. Consequently, the Ener_geter as a gamification element encouraged the participants to build more RE plants by targeting autonomous motivation through the gamification element [25, 26]. The Ener_geter embodies the idea of setting a concrete goal and enables clear communication, which is a prerequisite for acceptance.

’Vision:En 2040’ makes citizens aware of their responsibility in the energy transition process (see research question 2, introduction). In the focus group phase, the dialogue tool visualized the impact of their actions by using the Ener_geter to show the effect of a negative or positive attitude towards certain RE plants. For instance, if participants reject the allocation of wind turbines, the target electricity yield cannot be achieved. In their simulations, the participants always included discussions of the RE electricity mix and the storage of RE electricity.

’Vision:En 2040’ creates a space for dialogue processes (see research question 3, introduction). The dialogue tool provides participants with a discussion framework that they can use in a variety of ways in their simulation. All focus groups exchanged arguments for and against the placement of RE plants. In doing so, they integrated the target electricity yield and area suitability into their RE allocation planning. The qualitative evaluation also showed that the participants brought background knowledge, e.g. from their own RE projects, to the focus groups and that the focus group members integrated this information into the simulation of RE plants. Since focus group members incorporated the arguments of other participants into their statements or even changed them, we assume that social learning processes took place.

From the survey results we conclude that ’Vision:En 2040’ can broaden the understanding of different positions regarding the development of RE in one’s own municipality. The participants found ’Vision:En 2040’ helpful in finding renewable energy sites together. For them, ’Vision:En 2040’ has the potential to develop a common allocation plan for renewables in the municipality and to identify consensus areas for the allocation of RE plants (see research question 3, introduction).

’Vision:En 2040’ influences the evaluation and action levels [31] of acceptance of local RE allocation (see research question 1, introduction). The evaluation showed that participants of a ’Vision:En 2040’ workshop could change from silent to active supporters. For example, a large proportion of respondents to the pre-test and public workshop indicated that they would like to learn more about the topic in the future and become more actively involved in the energy transition. The focus groups further reinforced the participants’ positive attitudes toward the energy transition and the local siting of RE installations. The introductory presentation and the focus groups broadened and deepened the participants’ knowledge of the energy transition. People with more information and knowledge are more likely to support a transition to RE in their municipality [32–34]. Furthermore, participants indicated that ’Vision:En 2040’ will become a topic of discussion in their environment. The knowledge gained from the workshop can then be passed on. This could have the effect of reaching other people who did not directly participate in the workshop. In addition, the results of the workshop were presented to the municipal councils and influenced the development of a land use plan, which was adopted without further objection in a regular public participation process.

In general, ’Vision:En 2040’ is a dialogue tool that can support, but not replace, the official planning and approval processes for the allocation of RE plants. The workshop participants enjoyed the RE allocation simulation because of the integrated gamification elements. However, the scope of the ’Vision:En 2040’ workshop was limited to a maximum of 36 participants, and 24 attended. Therefore, the recruitment of participants should be reviewed and the possibility of increasing the number of participants should be explored. ’Vision:En 2040’, which was designed for use at the local level, can be transferred to the regional level or to municipalities outside the Hanover region if the database is expanded accordingly.

## 5. Conclusion

The majority of the population in Germany has a positive attitude towards renewable energy. But when it comes to siting wind turbines in their municipalities, there is skepticism and even resistance. Therefore, we designed and tested a new participation format based on behavioral mechanisms (’Vision:En 2040’) to reduce biases against renewable energy and to support informed decision making while taking local responsibility. ’Vision:En 2040’, a participatory format with a digital dialogue tool, provides knowledge about a sustainable energy transition and supports a cooperative simulation.

The dialogue tool includes gamification elements. It promotes climate-related action as a protected emotional experience space and provides a framework for cooperative learning and discussion. Evidence suggests that this participation also contributes to acceleration. The prerequisites for this are:

- Specification of the national climate targets for the local level, preferably in the form of local energy targets instead of area targets, in order to expand the scope for decision-making and allow flexibility in the energy mix.
- The definition of human- and nature-friendly spatial boundaries–the identification of suitable areas–for the sustainable siting of wind turbines and solar parks.
- Supporting the participation process with innovative participation tools that consider behavioral mechanisms to promote self-efficacy, collective responsibility, social learning and, last but not least, enjoyment of the participation process.

’Vision:En 2040’ is suitable for initiating an equal discussion process in municipalities regarding RE planning, promoting social learning processes, and creating acceptance. Evaluations showed that the dialogue tool made participants aware of the need to further allocate RE plants in the municipality. Their responsibility in the energy transition was emphasized as the dialogue tool showed the impact of a negative or positive attitude through gamification. ’Vision:En 2040’ broadened the understanding of different positions regarding RE allocation and was considered helpful in finding RE sites together. For the participants, ’Vision:En 2040’ offers the opportunity to create a joint RE allocation plan and to design their energy landscape.

An address-oriented manual should be prepared to implement a ’Vision:En 2040’ workshop in other municipalities independently of the research project.

## Supporting information

S1 Table Area suitability classes for wind energy on land. Classes of area suitability for onshore wind energy with their assigned surface categories and data sources.
(DOCX)

S2 Table Area suitability classes for solar parks. Classes of area suitability for solar parks with their assigned surface categories and data sources.
(DOCX)

S1 Text Excursus: Calculation of the area suitability classes. (DOCX)

S2 Text Excursus: The calculations of the potential electricity yields. (DOCX)

S3 Text Excursus: Calculation of the target electricity yield for each municipality. (DOCX)
