# Supplementary material for: Participation 3.0 in the implementation of the energy transition—Components and effectiveness of an interactive dialogue tool (Vision:En 2040)
Source: PLoS One. 2024 Mar 4;19(3):e0299270. doi: 10.1371/journal.pone.0299270 (PMC10911590; doi:10.1371/journal.pone.0299270)
Supplement: S1 Table — Classes of area suitability for onshore wind energy with their assigned surface categories and data sources. (DOCX) [file pone.0299270.s001.docx]

# S1 Table

# Area suitability classes for wind energy on land. Classes of area suitability for onshore wind energy with their assigned surface categories and data sources

| Wind energy on land (based on [50] and [49]) | |
| --- | --- |
| Area suitability classes | Land categories (Data set and data source) |
| Excluded | - Surfaces with a slope of ≥ 30° (Digital elevation model (DGM 50), LGLN 2019) - Water areas (BasisDLM, LGLN 2020) with riparian zone - National parks (NLWKN 2020) - Nature reserves (NLWKN 2020) - Bird sanctuaries (NLWKN 2015) - Natura 2000 network: FFH areas (NLWKN 2015) - Residential areas (BasisDLM, LGLN 2020) with distance buffers. Calculated according to the Technical Instructions for Protection against Noise for an immission guide value of 40 dB(A) (Walter et al. 2018). For the low wind turbine, a distance of 783 m from settlements, and for the high wind turbine, a distance of 842 m. - Industry/Commercial areas (BasisDLM, LGLN 2020) with distance buffers. Calculated according to the Technical Instructions for Protection against Noise for an immission guide value of 60 dB(A) [61]. For the low wind turbine, a distance of 78 m, and for the high wind turbine, a distance of 84 m. - Highways (BasisDLM, LGLN 2020) with distance buffers of 109 m (low wind turbine) and 120 m (high wind turbine); calculation: 40 m + (rotor diameter/2) - Federal roads (BasisDLM, LGLN 2020) with distance buffers of 89 m (low wind turbine) and 100 m (high wind turbine); calculation: 20 m + (rotor diameter/2) - Overhead power lines (BasisDLM, LGLN 2020) with distance buffers corresponding to the rotor diameter, here 138.25 m (low wind turbine) and 160 m (high wind turbine) - Cable cars (BasisDLM, LGLN 2020) with distance buffers of 415 m (low wind turbine) and 480 m (high wind turbine); calculation: 3 × rotor diameter - Rail routes (BasisDLM, LGLN 2020) with distance buffers of 199 m (low wind turbine) and 260 m (high wind turbine); calculation: hub height + (rotor diameter/2) - Air traffic (BasisDLM, LGLN 2020): Differentiation between airports, incl. distance buffers of 5,000 m, and airfields, incl. distance buffers of 1.760 m - Green Belt Germany (BfN 2013) - Military training areas & postmining landscapes, pits, and stone quarries (BasisDLM, LGLN 2020) - Wilderness and forest development areas (BfN 2016 and 2013) |
| Not suitable | - Ramsar-Wetlands (BfN 2013) - Biosphere reserves: core zone (BfN 2020) - Historical forest locations (BfN 2003) - 200 m buffer zone around sanctuaries (based on BfN-Data) - Heath, moor, and swamp (BasisDLM, LGLN 2020) - Landscapes with high landscape aesthetic quality scores (Hermes et al. 2018, IUP) |
| Partly suitable | - Biosphere reserves (buffer and transition zones) (BfN 2020) - Natural floodplains (HQ extreme, MU 2020) - Nationwide biotope network (BfN 2012) - Undissected low-traffic areas (BfN 2015) - Deciduous and mixed forests, shrubs, and additional vegetation (BasisDLM, LGLN 2020) - Landscape conservation areas (German cat.) (NLWKN 2020) - Buffer around recreation areas: 1,000 m (based on BasisDLM, LGLN 2020) - Coniferous forests (BasisDLM, LGLN 2020) |
| Suitable | - Grassland outside the area mentioned above categories (BasisDLM, LGLN 2020) - Arable land outside the area mentioned above categories (BasisDLM, LGLN 2020) - Landscape with lower landscape aesthetic quality scores (Hermes et al. 2018, IUP) |
