# Supplementary material for: Participation 3.0 in the implementation of the energy transition—Components and effectiveness of an interactive dialogue tool (Vision:En 2040)
Source: PLoS One. 2024 Mar 4;19(3):e0299270. doi: 10.1371/journal.pone.0299270 (PMC10911590; doi:10.1371/journal.pone.0299270)
Supplement: S2 Table — Classes of area suitability for solar parks with their assigned surface categories and data sources. (DOCX) [file pone.0299270.s002.docx]

# S2 Table

# Area suitability classes for solar parks. Classes of area suitability for solar parks with their assigned surface categories and data sources.

| Solar parks (based on [4]) | |
| --- | --- |
| Area suitability classes | Land categories (Data name and source) |
| Excluded | - Nature reserves (NLWKN 2020) - National parks (NLWKN 2020) - Biosphere reserves: core zone (BfN 2020) - Natura 2000 network: FFH areas (NLWKN 2015) - Water protection areas (zone I) (NLWKN 2020) - Riparian strips (BasisDLM, LGLN 2020) - Forests and woodlands, shrubs, and additional vegetation (BasisDLM, LGLN 2020) with shaded areas (BasisDLM, LGLN 2020) - Infrastructure (railways, roads, motorways) (BasisDLM, LGLN 2020) - Distance buffers of 40 m to highways (based on BasisDLM, LGLN 2020) - Water areas (BasisDLM, LGLN 2020) - Airports (BasisDLM, LGLN 2020) - Residential areas, recreation areas, industrial and commercial areas (BasisDLM, LGLN 2020) - Military training areas & postmining landscapes, pits, stone quarries, and dumps (BasisDLM, LGLN 2020) - Topography (slope inclination and orientation: all areas with a slope inclination greater than 45° and all north-facing slopes with a slope inclination greater than 30°) (Digital terrain model, grid cell size 50 m, LGLN 2019) |
| Not suitable | - Biotope network: Functional areas ‘forest’ and ‘semi-open landscapes’ (if not arable land) (BasisDLM, LGLN 2020; NLWKN 2018) - Landscapes with high landscape aesthetic quality scores (Hermes et al. 2018, IUP) - Swamp (BasisDLM, LGLN 2020) - Extensive grassland (NLWKN 2008) - Arable land (high to extremely high soil fertility) (BasisDLM, LGLN 2020; LBEG 2019) - Specialised crop pe orchards (BasisDLM, LGLN 2020) - Resting and feeding areas of wintering Nordic guest birds (Areas of the funding priority Northern Migratory Birds, NLWKN 2020) - Flood risk areas (MU 2020) - infertile land /vegetation-less area (e.g., riparian strips; BasisDLM, LGLN 2020) |
| Partly suitable | - Landscape conservation areas (German cat.) (NLWKN 2020) - Biosphere reserves: buffer and transition zones (BfN 2020) - Heath and moor (BasisDLM, LGLN 2020) - Historical Cultural Landscapes of Lower Saxony (NLWKN 2018) - Landscape with medium landscape aesthetic quality scores (Hermes et al. 2018, IUP) - Water protection areas (Zone II) (NLWKN 2020) - Bird sanctuaries (NLWKN 2015) - Arable land with importance for biotope network (BasisDLM, LGLN 2020; NLWKN 2018)) |
| Suitable | - Arable land (low-yield soils) outside the area mentioned above categories (BasisDLM, LGLN 2020; LBEG 2019) - Landscape with lower landscape aesthetic quality scores (Hermes et al. 2018, IUP) - Water protection areas Zone III A and B (NLWKN 2020) - Grassland outside the area mentioned above categories (BasisDLM, LGLN 2020) |
