# Supplementary material for: Participation 3.0 in the implementation of the energy transition—Components and effectiveness of an interactive dialogue tool (Vision:En 2040)
Source: PLoS One. 2024 Mar 4;19(3):e0299270. doi: 10.1371/journal.pone.0299270 (PMC10911590; doi:10.1371/journal.pone.0299270)
Supplement: S1 Text — (DOCX) [file pone.0299270.s003.docx]

# S1 File

# Excursus: Calculation of the area suitability classes

We used a geoprocessing workflow to assign spatially delimitable surface categories to the four classes of area suitability. The assignment of the classes of area suitability is based on three research projects [4,49,51] in which the classification was elaborated with stakeholders (Tables S1 and S2). We have incorporated the implementation of the National Biodiversity Strategy into the calculation of area suitability classes, for example by including wilderness areas [49]. Class delineation was implemented in a Geographic Information System (GIS) using the following steps:

1. collection and preparation of spatial data;
2. plant-specific calculation of buffer zones around infrastructure and settlements to include, for instance, noise protection in the classes (S1 Table)
3. merging of the surface categories into the respective area suitability class;
4. The feature classes of the area suitability classes are subtracted from each other according to the maximum value principle in order avoid overlaps in the final classes.
