# Supplementary material for: Participation 3.0 in the implementation of the energy transition—Components and effectiveness of an interactive dialogue tool (Vision:En 2040)
Source: PLoS One. 2024 Mar 4;19(3):e0299270. doi: 10.1371/journal.pone.0299270 (PMC10911590; doi:10.1371/journal.pone.0299270)
Supplement: S2 Text — (DOCX) [file pone.0299270.s004.docx]

# S2 File

**Excursus: The calculations of the potential electricity yields**

The potential electricity yield of a wind turbine is calculated from the product of the number of hours, the relative frequency of the individual wind speed and the wind turbine’s power in relation to the respective wind speed [64]. Initially, data on wind speed at 100 m above the ground was extrapolated to the hub height of the wind turbine. The extrapolation was performed by using the logarithmic wind law.

Log wind profile: $v\left( h_{2} \right)= v\left( h_{1} \right) \times\frac{\ln\frac{h_{2}}{z_{0}}}{\ln\frac{h_{1}}{z_{0}}}$

where *h*_1_ is the Reference height in m, *h*_2_ is the new height in m, *v*(*h*_1_) is the wind speed at reference height in m/s, *v*(*h*_2_) is the wind speed at new height m/s, *hz*_0_ is the ground roughness in m [64].

Subsequently, the relative frequency of the mean wind speeds occurring in Germany was calculated to intersect these frequencies with the power curves of the considered wind turbines. The Rayleigh distribution was used to calculate the relative frequency distribution (a_k=2_ and k = 2) [49,64].
$\left( v \right) = \frac{\pi}{2} \times\frac{v}{\bar{v^{2}}} \times exp\left( -\frac{\pi}{4} \times\frac{v^{2}}{\overline{v^{2}}} \right)$ ^22^

Enercon GmbH provided the performance data for the example turbines [50].

The potential electricity yield of solar parks is calculated in the dialogue tool from the area of the marked solar park in ha and the potential electricity yield of 1.09 GWh/ha per year [4].

The annual average electricity yield of PV on roofs is calculated from a potential area-yield value for Lower Saxony (potential annual electricity yield per area - TWh/a/km²) multiplied by the selected percentage of the usable roof area in the municipalities of the dialogue tool. The data was derived from an area and electricity yield potential calculation in the research project "EE100-konkret" [51].
