# Supplementary material for: Participation 3.0 in the implementation of the energy transition—Components and effectiveness of an interactive dialogue tool (Vision:En 2040)
Source: PLoS One. 2024 Mar 4;19(3):e0299270. doi: 10.1371/journal.pone.0299270 (PMC10911590; doi:10.1371/journal.pone.0299270)
Supplement: S3 Text — (DOCX) [file pone.0299270.s005.docx]

# S3 File

**Excursus: Calculation of the target electricity yield for each municipality**

According to a nationwide scenario calculation [50], 91.73% of the total electricity demand (1,500 TWh/a) will be covered in 2040 if all human- and nature-friendly usable area potentials for onshore wind energy and PV on roofs are utilized. In addition, 213 TWh/a are provided from geothermal, hydropower, and offshore wind energy. Since solar parks were not integrated into the nationwide scenario calculation, their installation can cover the lacking 8.27% of the potential total electricity demand or contribute much more if the ambitious energy-saving targets are not achieved.

To calculate the target electricity yield, we applied data about nationwide suitable areas for onshore wind energy and area potentials for PV on roofs of the nationwide scenario calculation. The delineation of municipalities, a table of electricity yields per federal state for PV on roofs and onshore wind energy [51], and the electricity yield potentials for solar parks [4] served as further data basis.

First, we calculated a TWh/a/km² value for wind energy and a TWh/a/km² value for PV on roofs for Lower Saxony. For this, we used the suitable areas and electricity yield potentials calculated of the nationwide scenario. The spatial data set with the municipality’s boundaries were intersected with the suitable areas for onshore wind energy and area potentials for PV on roofs of the nationwide scenario to determine the area potentials per municipality. The achieved TWh/a/km²_wind_ value is applied to multiply it with the suitable areas for wind energy according to the nationwide scenario per municipality to conduct a TWh/a_wind_ value per municipality. We used the same approach to generate a TWh/a/km²_solar_ value for Lower Saxony and a TWh/a_solar_ value per municipality in the Hanover Region for PV on roofs. Finally, the potential electricity yields per municipality for wind energy and PV on roofs were added. The addition was complemented with 8.27 % of the solar park electricity yield potentials [50].
